# Supplementary figures and images for: Virus-derived sequences from the transcriptomes of two snail vectors of schistosomiasis, Biomphalaria pfeifferi and Bulinus globosus from Kenya
Source: PeerJ. 2021 Nov 15;9:e12290. doi: 10.7717/peerj.12290 (PMC8601052; doi:10.7717/peerj.12290)

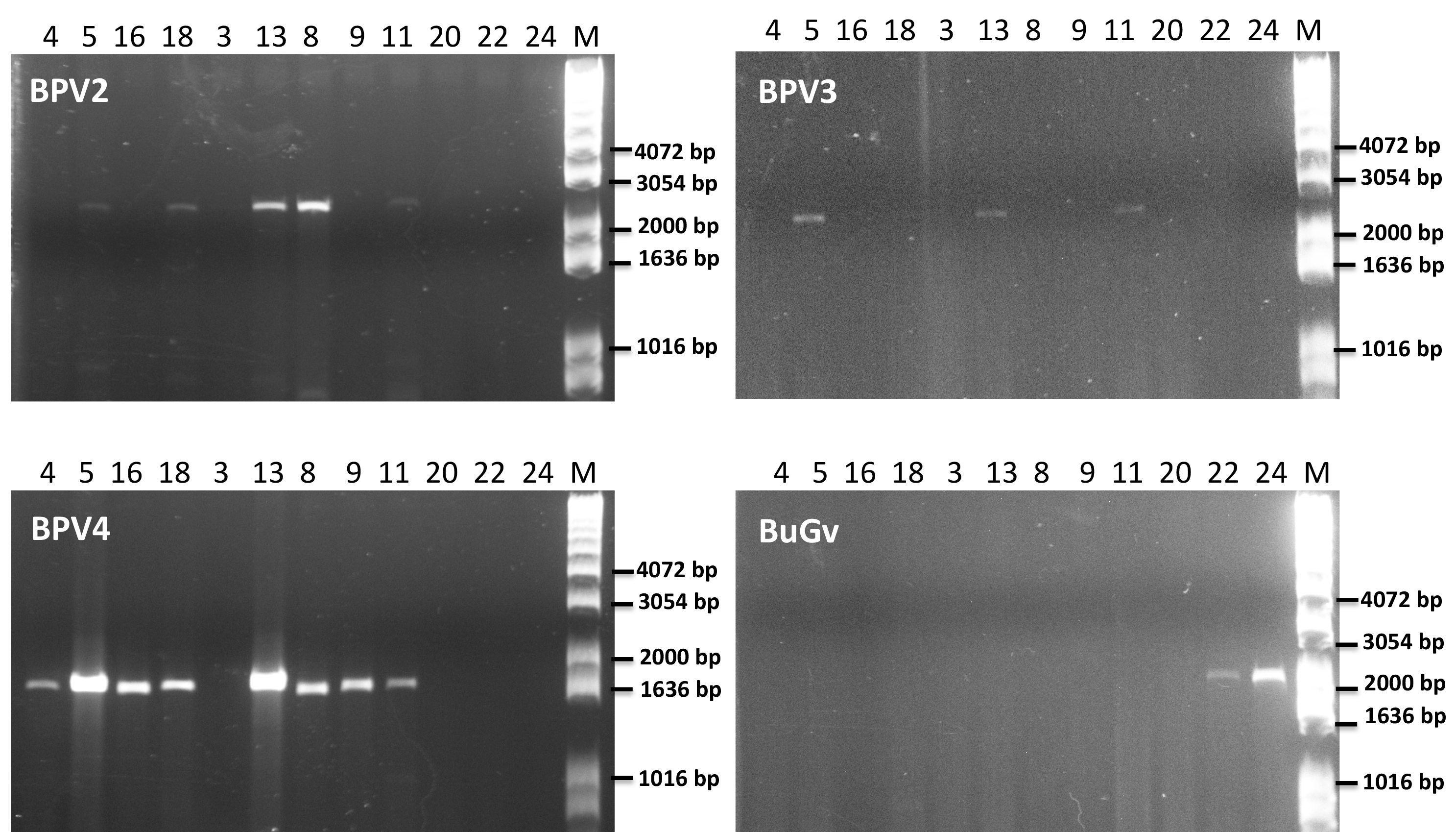

Supplement: Supplemental Information 1 — Long fragments of BPV2, BPV3, BPV4 sequences were detected in some BP samples, but not in BuG samples (22 and 24). BuGV1 amplification product was amplified only from BuG samples. Lane numbers correspond to sample numbers in Table 1. M, DNA size markers. [file peerj-09-12290-s001.png]
