# Supplementary material for: Virus-derived sequences from the transcriptomes of two snail vectors of schistosomiasis, Biomphalaria pfeifferi and Bulinus globosus from Kenya
Source: PeerJ. 2021 Nov 15;9:e12290. doi: 10.7717/peerj.12290 (PMC8601052; doi:10.7717/peerj.12290)
Supplement: Supplemental Information 2 [file peerj-09-12290-s002.pdf]

Supplementary Table 1: **Primers used for RT-PCR confirmation of assembled viral sequences**

Virus	Primer	Sequences (5'-3')	Location	Expected size of PCR product (bp)
BPV2	BPV2F1	GACCACGGAGGCATATCTATTG	7790-7811	339
	BPV2R1	GGTTATACGAACCCTGCCTAAG	8107-8128	
BPV3	BPV2F2	GCATATCAGGATGGCAGGATAG	5513-5534	2543
	BPV2R2	CTGGTAGAAGCAGCAAGGATAA	8038-8059	
BPV4	BPV3F2	CGCCTTTCGCTATCCTCTATTTT	1282-1304	2581
	BPV3R2	GTAACGCTTGCC ACTTTGATAC	3115-3863	
BuGV1	BPV4F1	TAGGCCACATAGTGCTGATTAC	619-640	1765
	BPV4R1	GGTTACGTGGAGGCTCTTATAC	2362-2383	
	BuGVF2	TGTGGAGACAAGCCGTTATG	3933-3952	2162
	BuGVR2	CGCATGAGACCGTAGTAGAAAG	6073-6094	
